# What is it all about? An explorative study of patients’ experiences with medication free treatment

**DOI:** 10.1186/s12888-024-06327-5

**Published:** 2024-12-02

**Authors:** Elisabeth C. Klæbo Reitan, Henriette Riley, Valentina C. Iversen, Anne Høye

**Affiliations:** 1 Division of Mental Health and Substance Abuse, University Hospital of North Norway (UNN), Tromsø, Norway; 2 Department of Clinical Medicine, UiT The Arctic University of Norway, Tromsø, Norway; 3 Department of Health and Care Sciences, UiT The Arctic University of Norway, Tromsø, Norway; 4 Department of Mental Health, Norwegian University of Science and Technology (NTNU), Trondheim, Norway; 5 Nidelv District Psychiatric Centre (DPS), St Olav Hospital, Trondheim, Norway

**Keywords:** Mental illness, Medication free treatment, Psychosis

## Abstract

**Background:**

As a response to the political decision by the Norwegian Ministry of Health and Care Services to establish some kind of “medication free treatment” for patients with severe mental illness throughout the country, a 6-bed ward unit dedicated to offer such treatment was in 2017 established in Tromsø, Norway by the North Norway Health Care Region. The aim of the present study was to explore the experiences of patients admitted to this ward unit.

**Method:**

Semi-structured interviews were conducted with 19 persons who had received treatment from the ward during the period January 2017 to October 2021. Analysis was done in line with Systematic Text Condensation and interviews were recorded, transcribed and analyzed using software NVivo.

**Results:**

The importance of engaging in a dialogue about the possibilities of living a life without medication was unanimously validated, along with a focus on empowerment, motivation, activity and flexibility. Not everyone reported fulfillment of their own wishes or the ward’s goal of tapering down, and reflected upon emotions such as ambivalence or fear. Three core concepts were identified to describe the participants’ experiences: 1) Tapering off, 2) Relations, and 3) Frames and content. A fourth concept overarches the process formed by these concepts; 4) Processes across categories.

**Conclusion:**

The study contributes to a deeper understanding of what "medication free" truly means, going beyond simply taking or not taking medications. It adds nuance to the debate surrounding medication free treatment. The ability to taper off medications is linked to intrapersonal factors, such as readiness and personal commitment, as well as the therapeutic environment, including the frames and values present on the ward. "Medication free" is more complex than it may initially appear, as many participants continue to use some form of psychotropic drugs. The sense of being part of something new and "exclusive" can be understood in light of what participants felt was lacking in previous treatment. It appears to be a need of rediscovering the significance of empowerment and empathic relationships in treatment of severe mental illness, in order to foster a sense of coherence and meaning.

**Supplementary Information:**

The online version contains supplementary material available at 10.1186/s12888-024-06327-5.

## Background

Long-term treatment recommendations for severe mental illness typically encompass a broad spectrum of approaches, combining both non-pharmacological and pharmacological elements[1]. Psychotropic medications are commonly considered a key component and recommended treatment in the long-term management of severe mental illnesses such as schizophrenia and bipolar disorder [1, 2]. However, the long-term use of psychotropic medication has also been questioned [3–5].

In the extension of this debate and with persistent input from user organisations, a novel treatment option offering patients with severe mental illness a possibility to manage without medication, was established by the Northern Norway Regional Health Authority in 2016 [6]. The goal of the establishment was to (authors’ translation into English) *‘’increase patient’s freedom of choice and expand the treatment offer in line with the core values of quality, safety and respect’’* [7]. The treatment was intended for people from the region who suffered from psychoses or bipolar disorder. This was in accordance with the document from a joint association of user organizations (Fellesaksjonen) [8], and governmental order [9]. The establishment was anchored in a state approved protocol and resolution [6]. Treatment is to be voluntary and in line with national guidelines. All four Norwegian health regions established medication free options, but mainly embedded in other treatment facilities, not as separate treatment units.

The medication free ward at the University Hospital of North Norway (UNN) in Tromsø is a separate, six-bed unit aimed at people with psychoses and/or bipolar disorder. Tapering down psychotropics is planned according to evaluation of prior experiences, availability of network support and emergency plans, status on basic needs (nutrition, sleep, rest and exercise), social factors (housing, income, activity) and social support. According to their philosophy, the ward represents a relational- and network perspective where mental conditions are understood as reactions to lived life rather than medical conditions. Consequently, treatment is not to reduce, alleviate or curb symptoms, but to deal with challenges (C. Nyquist, personal information, April 26th 2022 (Additional file 1)).

The importance of self-empowerment for medical adherence in severe mental illness has been described [10, 11]. Shared decision making goes, however, further than medical adherence [12–15], and is closely linked to the concept of recovery [16–22]. The ward’s description seems to align with Badu et al. [21], who describes recovery as a continuous, personal process rather than a clinical perspective focusing on outcome. Leamy et al. presented in 2011 a flexible model of personal recovery based on connectedness, hope and optimism, identity, meaning and empowerment (CHIME) [22]. Mueser et al. have identified psychoeducation, recognition of early relapse warnings signals, development of coping skills for persistent symptoms and strategies for medication management as important recovery domains [23, 24]. Lean et al. added a recovery focus based on personal goals [13]. The importance of personal recovery facilitated by psychosocial treatment is described also from other Norwegian health regions where medication free treatment is organised differently [25, 26].

The Norwegian Directorate of Health addressed the legitimacy of medication free treatment in light of concerns regarding patient consent competence [27]. Still, the mandate from the government to all healthcare regions triggered significant debate [28–36]. The main proponent of the directive, the user organizations, stressed the importance of individual autonomy, underscoring the right to make personal health choices [8]. Yeisen et al. highlight the scepticism regarding the basis of this decision, citing a lack of scientific backing [35]. In other health regions of Norway, medication free treatments were integrated into standard bed units, yet this approach also presented its own set of conflicts. Ødegaard et al. [37] discuss the dilemmas faced by therapists under the new policy, such as reconciling patient desires with treatment standards, available resources, and legal constraints. The discussions in professional communities now seem not to focus on whether non-pharmacological interventions per se are effective, but rather whether it is appropriate and safe to exclude pharmacological interventions.

The majority of the patients seeking treatment at the medication free ward have spent many years attempting to manage severe mental illness, primarily psychosis, through various treatment approaches. Most of the patients have used some form of psychotropic medication. Patients’ motivation for applying for medication free treatment is driven by a salutogenic hope for change, closely linked to the concept of recovery, as described in a previous study [38].

The purpose of the present study is to explore the experiences of patients who have undergone treatment in a medication free ward. It aims to gain insights into patients’ perceptions of non-pharmacological treatment approaches and to identify the unique characteristics they attribute to their experiences with the treatment. By examining these perspectives, the study seeks to offer new insights that potentially may offer valuable information considering both conventional and alternative treatment strategies. To the best of our knowledge, there is no research from other countries on comparable treatment options.

## Material and methods

### The medication free ward

The patient’s participation is emphasized by the phrase “nothing about me without me’’. This implies that self-referrals are accepted alongside referrals from local health care providers. Patients are permitted to add notes in their own medical record during the stay and are included in all meetings related to their treatment. The use of so-called “reflective dialogue” [39, 40] aims to strengthen the role of being an expert on one’s own life. The therapist’s role is to support exploration and the ability to make personal choices. If group activity is too demanding, individual treatment by an experienced consultant, psychologist, physiotherapist or psychiatrist is offered. Alternative activities are also offered. After dinner, the ward remains a community for both patients and staff together, yet it is also possible to withdraw. The treatment is preferably integrated with local health services. Admission frequency, length of stay and treatment focus are based on the patient’s wishes and anchored in network dialogue.

The staff includes trained nurses, occupational therapists, social workers, physiotherapists, psychologists, physicians and health care workers without specified education, and also includes user experience consultants. The weekly schedule from morning until dinner consists of five different activities: Recovery Workshop (RW), mindfulness training, creative workshop, recovery through music, and physical activity. The RW is inspired by the Illness Management and Recovery (IMR) programme by Mueser [23] and Recovery College [41]. Many of the thematic areas overlap with those found in the IMR programme, however the approach is less rigid, eschewing manuals and traditional lectures. The workshop emphasizes dialogue, present-moment situations, group reflections and the sharing of experiences from both patients and staff regarding the week’s theme (C. Nyquist, personal information, 2022, April 26th 2022 (Additional file 1)).

### Participants

Eighty-four patients were voluntarily admitted to the ward at least once from January 2017 to October 2021. Seventy-nine of them received a written invitation to participate in the study.

We have previously described the population of 84 consisting of 32 men and 52 woman who, at admission, were aged 18–63 years [38]. We were not able to contact five individuals due to missing addresses for four and one who was deceased. Nineteen persons signed the informed consent and completed the interviews according to the interview guide described earlier [38]. The majority were women (n = 14 of 19) ranging from 25–53 years of age. The median number of years with mental illness was 15 years (ranging from 8–28 years). Median number of admissions to the ward was 8 (ranging from 1–30). Hospital patient files provided supplementary information on admissions, treatment, diagnoses and medication.

All participants had at the time of admission a diagnosis of severe mental illness according to ICD-10 [42]: 11 had a main diagnosis of schizophrenia, schizotypal or delusional disorders (F20-29), 5 had Mood affective disorders (F30-39), 4 had Neurotic, stress-related or somatoform disorders (F40-48), 3 had other diagnoses (F60-69, F10) and 1 had no diagnosis. Several had secondary diagnoses. At the time of data collection, 14 participants had not had any diagnostic adjustment during treatment whereas 5 had their diagnosis adjusted.

### Interviews

The interviews were performed according to the interview guide (Additional file 2), in proximity to where the participant lived, at the site of admission or digitally. The individual interviews varied in time from 1:50 to 4:15 h. Sixteen interviews were performed in one session, three were split in two sessions. Interviews were recorded, transcribed and transferred to the computer-assisted qualitative data analysis software NVivo 1.6.1 after de-identification. The authors translated the citations from Norwegian to English.

### Procedure for data analysis

As described in detail earlier [38], Malterud Systematic Text Condensation (STC) [43] was applied. Inclusion of participants continued till saturation was considered achieved. Theoretical memos on ideas and associations were continuously written during interviews and the process of analysis started conducting the first interview. The content consists of both concrete (therapeutic approach and structure) and abstract (experience and assessment) expressions.

## Results

The treatment status at the time of the interview is shown in Table 1. At the time of admission, all participants had a history of using psychotropic medication. Although tapering off was described as an important aspect of their treatment motivation, the process proved to be more complex. This is illustrated by the fact that, at the time of data collection, 14 were still using antipsychotics, 3 were using mood stabilizers, 4 were using antidepressants, 10 were using anxiolytics and 3 were using other sleep-related sedatives (Additional file 3).

**Table 1:** Treatment status at the time of the interview (*N* = 19)

Still in treatment course at the ward^a^	5
Reported worsening of illness during treatment course	3
Had to stop tapering off medication	3
Quitted treatment at the ward by own choice	3
Had ended treatment according to plan	2
Quitted (due to other factors)^b^	3

^a^Four were admitted at the time of interview; two at the ward, two elsewhere

^b^Moving, lack of follow-up locally

**Fig. 1:**
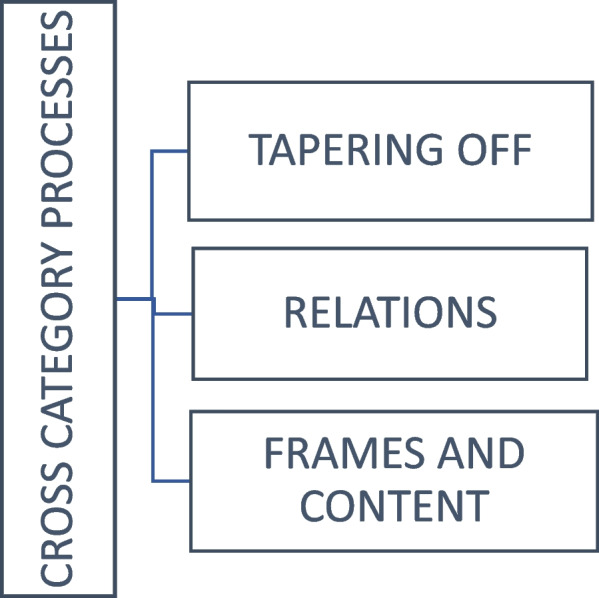
Concepts describing the experience of treatment at the medication free ward

Through interview analysis, we identified three main concepts, as shown in Fig. 1: 1) Tapering off, 2) Relations and 3) Frames and content. A fourth concept overarches the process formed by these concepts; Processes across categories.

### Tapering off

For most participants, the process of tapering off medication is a significant aspect of their treatment in the medication free ward. They share detailed accounts of their experiences, emotions and reflections associated with this phase. Additionally, they reflect on the process of tapering off, discussing their personal decisions and the steps they have taken.

#### Experiencing support

All participants express that they are grateful for the support on tapering down. Also those that have tapered down on their own appreciate the opportunity to get help trying to live a life without medication:

*I think it’s very good that they do not have focus on medication, when you have*

*said that you don’t want to have any* (medication)*. That they listen to you (18)*

This validation is independent of whether it works out well or not, as is the acknowledgement of the process not being straightforward. However, the continuous dialogue helps to illuminate the complexities and individual variations in the journey toward reducing or discontinuing medication.

*I was terrified of tapering off medicines … the new way of thinking was difficult to take in at first … I thought that I need medication and will become ill if I don’t use medicines. I was a little shocked that there were other thoughts on that … many asked me: Do you feel that you are affected by how psychiatry thinks about medication … I said yes … then I started tapering down … but stopped because I was so very afraid … It has taken some years for me to feel confident on this … being confident that this will be fine, tapering down … I’m still a bit confused on who I am and what I struggle with. But. I think this tapering down is very, very nice (16)*

#### Ambivalence

Some report experiencing an emotional awakening and gaining a deeper understanding of their true selves. Coping with the emotions associated with illness is a significant aspect of this experience. Additionally, there is the challenge of exposing one’s vulnerabilities which can be daunting yet essential for personal growth and building deeper connections with others. This process involves sharing aspects of oneself that are typically guarded, which can lead to increased empathy and support from those around them.

*It’s strange when you, somehow, have not been able to feel anything else than indifference for some years, and then it’s strange with lots of feelings. Both being happy … and sad and, yes … It’s like starting over again …Somehow, it’s a shame letting other people watch one’s tears … It’s locked in somehow, and I can’t unlock it … We are working on it … it might take some time (18)*

*The biggest change is emotionally … earlier, I didn’t recognize my feelings. For instance, I didn’t know how to be angry. But now my feelings are strong, as a human being, and if I read something on my mobile, or in an article, I can’t hide, I can’t hide anything in my face … you can read my face easily. That was not possible before, I had no mimic (3)*

There are descriptions of increased sensitivity, but also stories about not getting help to regulate new emotions:

*Before I started tapering down, I thought that this would be a frequently repeated topic they would ask me about. And helping me in the process with feelings … But it has hardly been anything … I have missed it* (the nature*) a lot, being close to it, feeling it and being fond of it. But now I’ve got much of it back … The moon and the stars, it’s amazing! (16)*

Some describe becoming ill during the process, leading to a halt in tapering off. One was hospitalised and prescribed high dosages of antipsychotics again:

*When I stopped taking medication I stopped sleeping and … I have just recently got some hold on sleeping … need something to sleep on, if not I’m not sleeping … I had decided that I would not have any medication, but in the end, I had to. Because I almost became psychotic (18)*

Several participants described negative experiences associated with the process of tapering off, such as feelings of danger. These feelings can manifest as heightened anxiety, fear, and a pervasive sense of vulnerability. This emotional turmoil often complicates the tapering process, making it not just a physical challenge but a psychological hurdle as well.

*In a manner of speaking, if it hadn’t been for the ward, I wouldn’t be alive today… I didn’t know how dangerous it could be to stop abruptly … that was what I used to do… I was not prepared for sleep disturbances, or getting heavy anxiety… losing sight of the meaning of life, and stuff like that. That’s where I am now, searching for meaning (18)*

Some conclude that tapering off is not the right thing for them, that it costs too much.

*I’m afraid of getting ill again … afraid of losing what I have accomplished … worried and get nervous that things will … fall apart… that everything I’ve been working for, will get lost (5)*

*I got my management plan put into my medical journal … I did get the possibility to quit medication immediately when I started seeing him (*therapist*). But, I did get ill within four months, as I always do … I did get what I wanted (*a chance and a management plan*), and I have tried to live without medications, and yes, it went poorly (7)*

For some, everything seemed fine at first, but then they later became gradually ill, e.g.:

*When leaving hospital last time (*having tapered off*): Then I felt that my mental health was very good. But then it started little by little, and then more and more, and suddenly it was … It took some months before I got really seriously ill…When I didn’t use medication, my thoughts ran fast. It went so fast. What the medication does; it slows down the fast thought processes … Brakes it, to work as normal … not be chaos (14)*

Some experienced a more sudden worsening of illness, and that this was not recognized:

*There was a risk that I might become ill, mentally ill or psychotic or something. At the same time, I felt that I was in control. That I could, if I felt for it, that I could increase medication or something. But, at one point or another here at the ward, I forgot this possibility of increasing medication … I don’t want to be negative … Because I’m a person on whom it is difficult to see if I’m ill … until I get really ill. Then everyone can see it… It was all gone, I didn’t remember that I could (silence) I didn’t remember to ask for an increased dosage (17)*

*For a while I was really pissed off at the ward because they had not been catching up on me … perhaps I would not have become as ill as I did (2)*

Several participants expressed feelings of insecurity and ambivalence about tapering off (e.g. 7, personal application). Some mentioned that the decision not to taper off was particularly difficult, partly because it could potentially exclude them from accessing other aspects of treatment at the ward.

*I wanted to stop taking medicines. Was very ambitious … I wanted other methods to handle my symptoms. And I feel I have achieved that but that it was not right to stop taking medicines. I have had to realise that I need medication … But I have learned that I’m sensitive to medicines… I think it might, in time, be right to taper off, or sigificantly taper down … having a small dosage makes a large difference … I feel that my quality of life is good. But I’m affected by illness and that I’m lowered* (in mood) *from medication … (15)*

*I have believed in the possibility of having zero medication … But I do have much anxiety and restlessness … I have believed that ending medication, it will take, it can easily have an effect on me … And I didn’t get ill, not in that way … like when I have been hospitalised … But I, I started getting problems, I started feeling* (silence) *more confused* (silence) *… then I took hold and started on medication again … But, the thought that pops up now and then, is that it is possible to live without medication (5)*

*I wanted to process a number of things and experiences … I didn’t know what I was getting myself into … At first it was not for me, the intention of tapering off, you know. … when I came I was using 850 and by now I have 300 (*mg*) … It’s my first real, conscious, tapering down* … *I’ve got a lot to lose, if (*silence*) If I become ill again … You feel much more secure if you do have a safety net (9)*

*I’ve been trying several times … it has not been right for me to quit taking all medicines. But I have the experience that using the smallest dosage is the best thing for me … Different from before, when it was necessary to have as much as possible. Almost (15)*

#### Hope and self-confidence

Many participants talk about experiences related to hope:

*I just saw a glimpse of hope that hadn’t been there for years, that maybe there is something else (4)*

*When you are down with bottomless despair and depression … they have been good at standing by at the time being … For the first time there is a tiny hope, and I have started looking forward, at least exploring, being very curious of what is possible even though it’s still quite heavy (2)*

Self-confidence refers to the belief in one's own abilities and judgment. It is an important aspect of our mental health and well-being, influencing how we interact with others, handle stress, and make decisions. Building self-confidence can involve various strategies such as setting realistic goals, maintaining a positive mindset, learning from failures, and surrounding oneself with supportive people. The connection between self-confidence and hope is described by several:

*Earlier, I was told that I was always going to have the diagnosis. You’ll always be needing help and for the rest of your life you’ll have to use medications … It was restrictions all the time … (*talks about the ward*) looking for options, all the time. My belief in myself, when I first entered … was really not existing, but then, for each admission, as they saw behind the diagnosis and medications and believed that I too, possibilities opened up … to be able to have a good life. That I could be able to manage by my own and to do the things that I wanted … Being able to work with people if that was what I wanted to, getting back self-confidence (3)*

*It was more holistically … that is, for me it was something that I had longed for without understanding it myself (10)*

Some describe the experienced change in perception as positive, others find it frightening:

*Simply have to learn feelings, learn reactions … I don’t have feelings, I must learn them … I must learn how to react … Now I have to learn it again because medication have pushed them away (talks about building bricks, daring to show anger, sorting out what to keep) Need to practice your self–respect (10)*

The concern that using psychiatric medication might lead to a loss of emotional sensitivity or make one feel "emotionless" is not confirmed by everyone:

*I feel that even when I’ve been having the worst medication and stuff, I’ve been able to feel joy, somehow, feeling … different things. Being a mother and feeling motherly love. I manage being present and go by (9)*

#### Dialogue about medication

The information on specific conversations about medication is limited, but some participants highlight challenges, particularly concerning the use of anxiolytics. These discussions are by some described as difficult. Additionally, there is a noted need for having someone else take a more active role in managing and controlling the decisions around medication, to alleviate the personal burden.

*At times I can take a little too much (*oxazepam)*, and that is because I have things that trigger panic attacks … I don’t feel I get help for that … when I was there* (ward) *and was on such a low dosage that made me feel worse, having more psychotic delusions … if I also were to keep me away from* (oxazepam), *it would be too much… I was met with: Perhaps you can try something else, a little mindfulness or … I thought it was part of their plan when patients were to follow up on how to relate to feelings … how to regulate them, and such … I was almost surprised that I was left by myself with this… I started missing the old way they* (the system) *used to work ... where I didn’t have to decide everything … and they could take some control … the best would be a mix … and that they can think what is best (16)*

### Relations

The quality of relations is linked to continuity and presence, such as being *Treated as a grown up. Even (*from*) the night shift *(2)*.* Fellow patients are recurrently mentioned, mostly as a resource; to have someone to share with and to learn from, apart from the staff. One person expresses: *I think meeting fellow patients is gold* (9). Some are explicit that fellow patients bring the most important input:

*You can open up on your feelings, don’t have to say much, you can listen … You learn very much, very, very much, listening to other patients. OK, is that how they have been, it’s like this for me too (17)*

*I have learned so much at the ward. Half of what I’ve learned is from the other patients … they open up for doing that* (talking) *about things that mean something, personal stuff, private stuff, how to taper down … I’ve never experienced this before. It used to be ‘hush, let’s talk about the weather’ (4)*

Being allowed to share also strengthens the reciprocity, e.g.

*When new ones arrive, my maternal feelings arise … (*me*) saying “just give it some days” (4)*

Some describe a fellowship characterized by a shared aspiration for change, encompassing a continuous process that involves both fellow patients and employees. This ongoing process seems to foster a sense of community and mutual support, enhancing the collective experience and facilitating positive transformations over time.

*Often you were admitted with … the same people that had been there before. Like a little family … exchanging experiences (8)*

*There was an ongoing conversation … sitting down together with therapists and they started telling about something … just sitting being a conversation partner … that is very good (5)*

*They are not just sitting in their own rooms all the time. They are together with us constantly. You build alliances over coffee … by being available, by being present. It makes you feel increasingly secure with them (4)*

#### Safety and equality

Being part of a team is for some a new experience.

*The first time I got there, I met my team. We sat talking and I thought: Do I have a team?! It’s not only that there is a team that is going treat me, but I do have a team (19)*

*I thought that it was the way mental health service* (hospital) *did work … that you, you are powerless and decisions are made above your head … but it was not like that at all. In the beginning that was very scary … I was used to having others taking decisions for me. Here I had to start taking actions by myself … I have to be active and participate in my own life … There* (ward) *I felt that I was my own person. I had not felt that before. (3)*

Some describe how the ward helped them develop social skills and recognize the needs of others. The importance of relating to others is emphasized in this context. This suggests that the environment is experienced as providing opportunities for personal growth in interpersonal interactions, fostering a better understanding of empathy and collaboration among individuals:

*It might be one of the most difficult tasks ever, seeing oneself in relation to other people … Then you have to have mirrors that function well around you… if you’re going to manage it… and you have to work systematically. I think … If you’re going to handle it 100% (11)*

*Where do I want the limits to be or … how social do I want to be. Or, I choose more myself (*silence) *I try to be a voice for others when I’m open about my illness (*silence)* I miss being an important person for somebody … I have noticed that I’m more sensitive than I thought I was (9)*

The employees’ role in feeling safe is often mentioned.

*You get to know the ones working there and become more secure in the ward, then more and more will emerge. When you feel secure, you can notice more … when you enter, you are nervous. And you get used to open up … Getting the same questions after three months, what answers would you get then … Then you are feeling secure at the ward (14)*

(Employees) *want to create safety, they want me to gain trust in them … Somehow this is their main goal. That I shall be safe. Be able to relax … they want you to have frames in your life: I was afraid of and didn’t dare to, to open up and talk … from driving around to the back of the building, almost turning around and driving back home … now I drive to the parking lot and walk straight into the building … I say hallo and I am cheerful and happy. Instead of being filled with anxiety and nervousness (10)*

A sence of equality, being listened to, and employees sharing from their own lives are brought forward. Flexibility, positive encouragement, and playfulness are other important factors for experiencing a fellowship.

‘*We cheer for you, you just have to tell what we …. what we can do’ (9)*

*Then he called me … the next day, saying: I think you are perfect for us … And I thought: You are the only one who has ever said I was perfect for anything … I didn’t know what it was I was going to, just had to seize the opportunity: Perhaps there is another life for me than what I have … I had no idea that it would turn out this good (laughs) (4)*

However, for others, the help offered has not been working out.

*I’ve seen how they are with other patients, and I think they’ve been fabulous. I didn’t manage it. I had feelings too, but I couldn’t express them in the ward. I did manage to express myself in another ward (17)*

Both personal experiences and formal education among the staff are important factors when describing competence.

*They (*the ward) *were more fearless, perhaps they are more experienced regarding psychosis. You must know what you are doing. I do understand that if you don’t have any experience …* (silence*) (12)*

*You were guided to the person with the competence you were asking for. When one occupational therapist could not answer, the person said: I’ll find someone else that has the competence to handle that kind of questions (14)*

Including other helpers, such as mental health organizations and a priest, is considered to be positive:

*That make them qualified to help differently from the ones who have their only knowledge through education … (8)*

Characteristics of employees are frequently mentioned as being important for one’s ability to change. There are of course individual differences, but some descriptions stand out:

*He is my wingman … tapering down means so much more, it’s a whole, whole, whole life and a whole new life … You have protection and care, eh. It means someone who give you courage to … and continues … when I manage to be secure on it myself, being secure on others … time for you to become that (10)*

Employees’ high levels of tolerance is underlined, and a focus on other measures than taking control. One participant describes being welcomed back after leaving the hospital after an episode of acting out: *They were professional, off course (11).*

*It was a very bad day … They didn’t get grumpy because of that (8)*

*If you were to cry, there would always be someone who could help … embrace you or follow you to the room. Yes, or doing something else to calm down (17)*

*What I like about the ward, is that you are seen… you are met as human being. They ask what do you need, what kind of milk you drink … And there is treatment … They do put the patient in focus… They look at your resources and what you can, make that a baseline (7)*

*Before it was focus on limitations … challenges and symptoms … I didn’t feel like that at the ward … It was: What are your strong sides and, of course, what are the challenges, but what can you do with that and from that, despite that (3)*

*I think it’s in the soul of it, showing confidence in patients and the person behind the diagnosis instead. Instead of trying to take control defining this is a psychiatric problem that we have to take control over with medications and prevent … They were good at handling the situation … regain a quiet ward again. Not just closing someone into the room (19)*

Promoting the individual’s control is frequently mentioned, e.g.

*I was in the driving seat, always … even when I was in bad shape … I felt that every decision was taken with the best of intentions … And that they made such an effort in getting to know me that they knew what I would wish for even when I didn’t manage to express myself … Nothing was done without me wanting it … At every admission it was like; what do you need, what is challenging now, how can we facilitate … Constant adjustment in relation to where I was … It didn’t matter what had happened last time, or what will happen in two weeks, but just here and now (3)*

*This was the first place that I have felt that what I got was proper help … Because they believed that one could get better … unlike being in storage… They were focusing on our resources … Yes, they do motivate us on daily basis (8)*

#### Giving feedback

Giving feedback to the ward is experienced differently. Some are satisfied, for example, stating: *There were always conversations about what could have been done better (8).* However, others feel misunderstood or overlooked:

*There is support, and not support … not all conversations are (*silence*) taken. They do express clearly that they want feedback, they need feedback, that the ward is new and developing … 75-80 % support and 20 % … things that remains (10)*

*I felt that I was alone and not heard. I didn’t shout load enough, you know. I didn’t slam the doors … I felt you were not seen the way I was hoping for (13)*

Some attempted to communicate with the staff after experiencing conflicts, but did not feel acknowledged. For example, one person expressed disappointment during a telephone conversation, saying *I did call saying that I was very disappointed over this and that … but I did go back (1).* After being discharged, this person requested a reconciliation meeting but felt that the concerns were not adequately addressed. There are other examples where communication was perceived as difficult or challenging:

*I felt that when I gave negative feedback on my therapist … then I started getting trouble with the ward … I was not allowed coming there. All the time, we had to address that they misunderstood what I said, that I misunderstood them … They said they would take hold of it, but. I felt that they didn’t (17)*

*They somehow had let me down and had not seen that I was psychotic (11)*

*I think it’s very positive that they challenge me to stand in the panic attacks, it’s very nice. But for me, when I’ve been there* (ward) *I’m so tired that somehow, somehow it becomes too much… perhaps there is too much focus on medication free … I have not spoken to anybody about that, but I feel that I’ve been so tired in my head that I simply have been exhausted … And I’ve tried, I phoned the ward … talked to them, because often I’m at a breaking point thinking I need help now. And I don’t get help (16)*

#### Unusual boundaries explored

Many found it surprising to encounter staff members who openly shared their own personal struggles. This level of openness and vulnerability from the staff was not what they had anticipated, but some participants mention that this positively impacted the experience, likely by making them feel more understood and less alone in facing their own issues.

*Several of them had experiences too … We talked together on the same level, they were not … on their high horse … like some psychologists and nurses who might be not very private, but you can talk to them, like people (15)*

Equality, or flat structure, is also reported as positive by many, e.g.

*The employees treated me, or they shared, shared their life, so there was really a cooperation regarding trust … I didn’t feel much inferior. There will always be a balance of power in this kind of relations … but I felt I was more human and not a patient … more nice conversations when the other person not only listen but can be normal … They have been working to gain my trust (3)*

However, others find this more problematic:

*I felt the person became a little too private for me … Person asked If I didn’t understand how hard it was for this person … It was just too much for me … it should be the patient’s choice if wanting to listen … And if the patients then say ‘no thanks,* no’ *so, ok. Don’t do it! … many do complain about this… that they get to hear much, that is shared. Many do not dare to make complaints about it … they just listen (17)*

#### Collaboration

The connection between the patients themselves, the ward, and local services are important. Some, but not all, have an established professional network that collaborates with and supports their private network.

*It’s important having some on your side … someone cheering … especially my parents … not everyone who suffer with mental illness have good support from their parents (8)*

*We made a network picture … but did not work on it more … I guess I have some work to do there … I’ve chosen that they (*family*) don’t have to know everything, because they don’t understand* (silence) *and it’s ok … then they don’t become the supporters that I could wish them to be … I do have many friends and acquaintances … but for those close things, I don’t have somebody (13)*

Network meetings mostly worked well, but some report that they are non-existent. Some describe regular meetings, others felt no need to involve the network more frequently.

*There is my GP, my spouse … my boss, my therapist at the local hospital. I used to include my parents but have stopped doing that because I want to protect them. I don’t want them to feel sad … I choose, can choose a good friend if I want to … They’ve said that it’s not often GPs take their time coming to these meetings. But he’s always joined (4)*

*They talked about strengthening my network and encouraged me to get in touch with friends and stuff … but it was not that I wanted to focus upon … it was not the most important thing for me at the time (15)*

A professional network continuity significantly enhances the experience of support:

*Unfortunately, we’ve experienced that we’ve not managed to cooperate. Therapist has quitted, no follow-up …* (the ward) *is the only one, the only one I have right now … It’s not an ideal situation … On paper they* (local health service) *are there … It was the local therapist who made application for me* (to the ward)* before she pulled herself out (2)*

*We’ve had network meetings almost every time I’ve been at the ward … I want everyone who has to do with me, to participate in these meetings. Then I don’t have to use time meeting them except from there … When you are ill, you are not good at using it* (network) *because you have more than enough with yourself (*silence*) I have many who (silence) matters … Someone from the municipality is to participate too, it has never happened before, so (*silence*) I’m excited about that (18)*

The professional network also has an important role for social life, through creating and fostering bridges between professional resources and personal lives and to alleviate the personal pressure on family and social life when a person experiences mental illness.

*Now I’m getting the help that I need … me and my cohabitant have gotten much closer … I used to scare him (6)*

*I feel that I have mental health service for that … and do not want to push my problems onto my people … I also feel like this thing with mental health is so private, that I do not want to call a friend saying that I’m struggling mentally … I do have a friend who has also been at the ward, I feel we can understand each other and can talk about it. It is very nice (16)*

*She* (child) *is allowed here … we are having network meetings with my psychiatrist … members of my family … friends … employer … a supportive network ... If they see change, they are allowed to tell (9)*

*They* (family) *love the ward (*laughter). *I’ve got, not only have I got, but my whole family has got, life as a gift, again … When I came I think I was on 700 mg (*clozapine). *At Christmas time I was on 100 … my daughter said: Mum, I don’t understand that you are to taper down anymore because you are so fine by now (4)*

### Frames and content

Frames, such as maintenance or improvement of circadian rhythm and defined schedules, are examples of what is reported as important for therapeutic change.

*You enter a new everyday life when you are to be up and fit for fight at nine o’clock in the morning, and preferably you get up earlier to eat breakfast … Just doing this, was very hard in the beginning … Because you are used to sleep long because when you are on medications you sleep for long … I used to sleep for 18 hours a day … But you need to have will. Being strong willed and have faith in it (18)*

*It was very good being met with some demands like the morning meeting at eight o’clock a.m. I myself needed it to prepare myself for getting back at work (4)*

Even so, flexibility is frequently mentioned:

*It changed to five-days-admissions because I didn’t manage to stay at the hospital for long … For more than a year I was there very often, once every month, once every sixth week, staying for five days. Then we started talking about me not going so often … Haven’t had many admissions the last years (16)*

The experience of noise in the ward differs. Some were bothered, e.g. *I didn’t get any sleep at all before … and then I didn’t get to sleep there either … because of a noisy patient (1),* whereas others were impressed with how unrest was handled:

*You would think that people with psychosis and mania, who are tapering down medication, that this place would be a place filled with unrest, but first of all, there is extreme acceptance and understanding between the patients … And if you are feeling bad you’ll be followed tightly, too, instead of being medicated … They can handle that you are feeling bad, or they help you. They do help us through so that we actually can get over, get over without using medication, and perhaps bring along what was the trigger. What it is about. Unfortunately, it takes very long time (2)*

#### Physical conditions

Some experience difficulties related to the location (the ward is located inside the hospital):

*Perhaps it should not be located here … should have been a house of its own down by the sea … because the ones who come here are so tired of the system, we want something else, want to be free from the risk of entering the building … the fear we get entering … or just seeing the building (10)*

One person talks about the architectural design providing opportunities for both interaction and privacy:

*A place for gathering. Some can play games while others talk in a corner. I think the architecture has helped … having these rooms for being together. You can go to the living room, or withdraw if that’s what you need (19)*

Some underline the physical appearance:

*I like that they have a living room with flowers and pictures (16)*

*It was cosy and the employees could play instruments, one of those working night shift played the guitar for me, and sang for me. I think it was very cozy. Something I never thought would happen in a ward (7)*

#### Diagnoses

Diagnoses are mentioned to be of importance in two ways. First, getting a diagnostic evaluation, which for some led to change. Second, *not* to focus on diagnoses.

*When you have been ill, you are the diagnosis … you dig yourself down, when they remove it, you immediately feel better because you are not the diagnosis (1)*

*They are not so concerned with your diagnosis … This kind of things (silence) I think they’re concerned about you as a human being … and what you want… for future life (9)*

One person whose schizophrenia diagnosis was removed, expresses:

*I was seen as being the person I am. My need … Not the diagnosis I had … For me that was the most important part of treatment … I had been told I would always have this diagnosis and I would always need help ... At the ward they looked at it differently. Looking behind diagnosis and medication (3)*

Some want to have their diagnosis changed, but had not achieved it yet:

*I still have the same diagnosis, I have tried to get it changed (*Second opinion) … *But next time at the ward, we are going to start talking about diagnosis … It’s not right, I have never felt psychotic (18)*

#### Treatment content

The participants primarily discuss their personal experiences with treatment rather than the specific details of the treatment itself. Very few present a clear description of the treatment content.

*The most important with this treatment … being seen as the person you are, not the diagnosis you enter with … it would not be what it is without everything … because if I hadn’t had recovery workshop I wouldn’t get so much from art therapy … a red line … getting deep into different themes (3)*

*I think there was more treatment there than at other places I’ve been admitted to. Recovery workshop, art therapy and conversations individually and in my group … I’ve been receiving art therapy other places too, but not so systematically (11)*

Elements most frequently mentioned are the recovery workshops, art therapy, conversations, physical activity, network meetings, crisis management plans. In recovery workshops, everyone can suggest a topic and participate. Some choose when to enter the ward dependant on the week’s topic:

*There are some weeks named something like “voice hearing week”. It’s been very nice because I hear voices … It’s very nice when all in recovery workshop are voice hearers. We’ve had the theme at recovery workshop when some were and some were not hearing voices. It’s very difficult for the ones who do not hear to understand how it feels hearing (18)*

In the workshop employees also share their experiences:

*When we have recovery workshop, the one who work there also share, many have experiences with mental health, experiences and stuff they think have been difficult (18)*

Art therapy is also mentioned as positive; doing something together, being able to talk and have a nice time, but also as something that enforces insight and thereby change, e.g.

*In creative workshop I feel like being 10 years old … not managing to draw. But they say: Have you tried painting? I say: No … Then we had an evening painting … Just sitting there, talking, listening to music and suddenly! (9)*

*If you try doing it at home, over time, you can reflect about it in the same way as in the art therapy sessions at the ward … it might help expressing oneself about oneself. Talking with oneself the same way one did talk with the group at the ward. Then it can be a tool … having an inner monologue. Explore by yourself a little (15)*

#### Individual adjustments

Physical activity is often discussed primarily in terms of its relationship to treatment rather than focusing on the specific content or types of exercises involved. People's experiences with physical activity as part of treatment can vary widely. However, the importance of flexibility and tailored facilitation is consistently emphasized.

*In the beginning when I came here, I hated physical activity … today I can walk all around the house … expand little by little … Then, coming here, they found the reason why I did what I did. It was almost magical … I’ve been lying on the sofa not feeling any happiness, no sadness. I’ve got back the laughter, the tears and … That is so funny! (laughs) (4)*

*If I said that today I can’t take it … it was ok, but. There were such an undertone saying that you should have … But I think they should have entered it more with a question why I didn’t want to, or find alternative ways for me to be physically active (*silence) (*3)*

However, being met on physical health was more than physical activity.

*There was a focus on eating what the body needs, or eating in general. Focus on physical activity too. The consciousness around this was different than before … One important thing happened at the ward: Side effects for the heart, symptoms or side effects related to heart disease were followed up for the first time* (neglected before?) *Yes, definitely (3)*

Some had to adjust their physical activity after tapering down.

*I used to be lucky to be in good shape, before I got ill, that is. Before I was admitted to acute ward I was training like an athlete (*laughs)… *After having lied down for five months (*acute admission*) without eating or drinking, losing 40 kg … I woke up again, started to walk and do some exercise. Not like before, my muscles were, you know … My body and my brain were exhausted from being so depressed … I’m getting so sad about it because physical activity is so important to me. Being in motion every day. I hope I’ll get there (17)*

*Physical activity is very, it’s what I have been using my whole life … For a while after tapering off, I was jogging every morning* (laughs*) … Because I didn’t sleep… But I have had to taper off much physical activity because my body gets too tired. I guess I stretched the elastic a bit too far. So, what I’m doing now, is doing a little bit less every day … Must find out how far my body goes (18)*

Plans for crisis management are considered important for many patients, as they help in managing potential emergencies effectively. These plans should be tailored to address the specific needs of each person, ensuring they receive appropriate care during a mental health crisis. Many express that working with a crisis management plan has been beneficial, e.g.

*Stabilisation and development after tapering off ... we made a management plan for crisis at the ward, for doctors and all … It took some time for me to approve it, but then I realised it was best if people got to know, the closest ones and the doctors (14)*

### Cross category processes

In the course of investigating the three preceding concepts—tapering off, relations and frames and content – there are associated processes that relate to these concepts but also stand as distinct subjects in their own right. Some overarch the three concepts, such as intrapsychological or personal development linked to adjustment of expectations, feelings connected to having a mental illness, and interpretation of personal challenges.

*I have been better at handling having hard times. But I must admit that I did join the treatment method with a little too high expectations … thinking that one would become completely healthy* (silence*). For me, that was not realistic (*silence*), to get completely rid of the symptoms (8)*

*I said that ok, I just have to choose to trust … the therapist … But I have somehow experienced that I genuinely can … trust them. Although it’s not always that easy. Yes. It’s been an issue for me because I have problems with relations. And I know there has been made much effort in making it safe (2)*

*Yes, I think I will be there* (in the process) *the rest of my life (4)*

#### Evaluation of the ward

There are catchy statements to describe what the ward is, e.g.

*Everything else has been like driving a Lada … I used to say; I’ve tried the Lada model, and I’ve been in a Porsche (9)*

*I’ve been so lucky that I have been admitted to the ward. It, it’s almost like winning in the lottery … like, it’s better than winning the lottery (4)*

Others are more critical*:*

*It’s not a special ward. It’s just another ward at (*the hospital*) ... My opinion is that there were people there who were too ill … and that they (*the staff*) demanded too little from them … I think, if you’re not able to get up of bed in the morning and go to breakfast at nine o’clock, brush your teeth, get … dressed. Then you’re not ready to be medication free (13)*

Many of the participants talk about how society perceives mental illness and how the option to choose treatment without medication could be influenced by a possible change in zeitgeist toward greater humanity and patient involvement.

*I do feel that it’s part of a positive trend in psychiatry … not just being stowed away … Being allowed to decide if you want to take medicines or, if you, what you want to do that particularly day (19)*

How people first heard about the ward differed (Additional file 4). For some it was easy: *I didn’t have to convince anyone … it didn’t take long time (14),* whereas others experience having to fight to get the possibility:

*They* (local hospital) *didn’t know about it … and it was written in my medical journal that I was delusional, having delusions about being part of a group, being paranoid talking about the ward (12)*

*You’ve got to have resources to find information. (I) got information through the website and I knew the people who built up the service (9)*

#### Mastery and recovery

There are reflections on who may benefit from this treatment:

*It can be for anyone, but you must, you must want to do something. You must work with yourself, your thoughts, and not being afraid of meeting some feelings (18)*

*The time I’ve been at the ward, I have been in a good period … And I do think that to make plans, being able to work on network, working with yourself and how to develop yourself and how to learn to live with the illnesses, then you have to be stable. I think of a ward for those who are stable, even though it’ s not like that … I think it’s best being there being stable (7)*

*You must carry some calmness inside, otherwise it will spread out in the ward (19)*

The opportunity to handle secondary emotional consequences of illness, such as shame and worries, is valued.

*Working through all the things that has not been good, and now being able to say: Yes. I’ve done that. I was so ashamed. And shame is so bad because it’s so difficult to handle (4)*

*Before, nobody understood, told me that feelings are felt in the body. They called it tension … Nobody said feelings are felt, that when you are afraid you feel it in your stomach. I didn’t know, I just had stomach pain (12)*

To master and to be in charge are important themes in many stories. One person says:

*Before I was sitting in the back of the room, in the corner, not saying what I thought and meant … didn’t really have so many thoughts about what I meant, wanted or such, either… Today it’s the opposite… I’m sitting in front, saying what I think and mean. Before I wanted people to like me… no I don’t really care … My family says they wouldn’t believe that I could develop so much ... Now I’m interested in humans… politics, human rights, eh, equality (3)*

Acceptance of illness, life and attitude towards oneself is frequently mentioned.

*You have to be honest with yourself and have faith that it will work … Accept yourself (14)*

*I’ve become much more well than I was before … I’m not satisfied with how I’m doing right now … with where I see that I might go. And where I hope to go (15)*

*It took some time for me to accept that… I’m not, I’m not bipolar. I have bipolar disorder. It’s part of me … and there is room for that … allowing, accepting that it’s like that … You just have to learn living with the illness … It’s part of my life, right … It doesn’t rule my life, but is still part of my life (13)*

For others, the focus on mastery was too strong:

*First time I was there I sat in my room for three weeks, shivering and being scared, terrified, so… But by now I dare to take space, and sometimes perhaps a little too much … they are really into individuality… in the beginning it was almost a little bit too much of self-determination (*laughs*)… It’s one of the things they are on … they want us to, that we should set some boundaries (2)*

*I think they wanted me to understand what autonomy was, somehow, that I had a right to vote for myself … the first time I got there, they gave me information about that I was not admitted with coercion… that was simply (silence) …. was a new way of thinking (11)*

The process of recovery is described by many.

*To re-cover yourself… I feel that the main focus was on getting better … Not necessarily getting well, but learning how to handle symptoms … finding tools and get to know yourself and by that getting to know the illness (15)*

*When you know what you want, you’ll get help. Set goals and you’ll make it. The therapist is best listening to you, he shall share his knowledge and show you the way, but you’ve got to walk together, cooperate. Everyday ... Recovery is you and what you need. No talk about deadline and money. Treatment are tailored to fit you. Recovery is different for you and for me … working toward getting it better (7)*

Differentiation and adjusting needs are mentioned. E.g. one person who is still in treatment, who describes severe inner pain and suicidal thoughts and the importance of mental preservation:

*It’s not that I want to kill myself, but I want peace … I feel that I have so much pain inside my body that eats me up … yelling in my head, voices. All the impressions that I’m not able to process … I’m afraid of getting ill again … It was very difficult (being in focus)… In the beginning I only managed 5, 10 minutes. Then all my energy was gone … By now it’s ok, I can sit one hour and talk about stuff (18)*

#### Motivation

Personal motivation is a recurring theme. Specifically, the personal motivation is emphasized as crucial for recovery, but it is equally important for the staff to not only be self-motivated but also proficient in fostering motivation among the patients. This dual approach ensures that patients feel supported and encouraged.

*Motivation is important, they try to help with that … I feel more heard … for the first time in my life I’ve started to stand up for myself and … They’ve tried to make me secure, all the time I’ve been here, that they will not throw me out before I'm feeling goo d… Since I experience change, then I believe in it a little bit more, that it is possible (2)*

*I think it works because I feel, I feel that, when you go there, it’s not enough to show up at the ward saying you are to live without medication. You need to be motivated…because it’s work all day… You need motivation, also to benefit from it… If you enter and think that, no, I don’t want to go for a walk, I don’t want… therapy, I find it boring. Then I don’t know if you have anything to do there (4)*

## Discussion

The results of the present study show that treatment at the medication free ward include both positive and negative experiences with tapering off medicines, but also important aspects such as self-empowerment, relations to others and frames needed for a safe recovery. The findings are closely linked to the participants’ motivation for applying [38]; an experience of mastery, a search for coherence, and comprehensible empathic frames and relations.

### Tapering off

All participants are positive to the idea of medication free treatment, also those who express negative experiences. They also express an analytical understanding of the continuity between idea and reality, and a gratitude for having been given the opportunity to try. Being met with a dialogue on the topic was a new experience, as was the feeling of not being alone in the process. For some, this dialogue was already going on locally before admission.

There is a more or less implicit understanding that medication side effects make life more difficult. This is of course partly due to the selection of participants. Still, some express that emotions are not absent when being medicated; such as feeling love and care as a parent. Also, not focusing on symptoms, but still having someone to talk with about medications, symptoms or side effects, is important.

#### Self-empowerment

Self-empowerment includes being allowed to set the agenda and decide whom to cooperate with. Empowerment also depends on health care professional’s belief in what they do [14] as well as the relation between patient and professionals [25].

The results in our study support the findings from Standal et al. [26] that simply being able to talk about medication is appreciated. Our participants describe the experience of not being alone, and learning from others. Also, to be met with respect when addressing whether medication is necessary or not, and to be given alternatives, was long awaited. However, some express that it might sometimes be “too much” having to decide by oneself.

#### The concept of recovery

The elements of recovery described by Leamy et al. in the CHIME model (connectedness, hope and optimism, identity, meaning and empowerment) [22] seem to align with our study. However, the link between outcome and recovery is subtle. Outcome is often described as positive goals like getting something to do, making family life work, and avoiding side effects. Outcome works as motivation, and the focus is the personal process. Several have underlined the need for flexibility, as also described by Leamy [22]. This is in line with Standal et al., who conclude that reflexiveness is important in how clinicians understand individualism-relationism, because people react differently to increased demands [26]. At the ward, there is a strong focus on non-medical interventions i.e. art therapy, physical activity, recovery workshop, fellowship between patients and between patients and employees. This is in line with recovery-based interventions as described by both Lean et al. [13] and Mueser et al. [23, 24], but with theme-based recovery workshops instead of structured psychoeducation or medicine management.

### Relations

Feeling acknowledged and supported, rather than being left to navigate the process alone, is closely tied to all the core concepts described. Being seen is particularly connected to the ability to make changes through emotional management and the exploration of new experiences. Positive elements such as flexibility, cooperation, belonging, fellowship, care, compassion, and being empathetically challenged all contribute to the process. The freedom to make choices appears essential, as does the opportunity for activity that fosters a sense of autonomy and strengthens the experience of oneself as an independent individual.

As articulated in Antonovsky’s theory on salutogenesis, a pivotal question is “whether it is worth it” [44]. This question is embedded within the expressed sense of coherence (meaningfulness, comprehensibility and manageability). Our findings support that this remains valid. However, while we have previously [38] reported a fragile sense of coherence in line with Bandura’s findings [45, 46], the experience of being at the ward presents a somewhat different scenario. We observe a dilemma in how this is expressed and attributed. When participants conclude that it is not worth the risk to taper off or live a life without medication, they often attribute this to personal factors such as not being able to do it, not being ready for it, or needing something else. There are few voices claiming incompetence or therapeutic shortcomings by the staff. Still, several report it as difficult that they had to “give up” the rest of the treatment “package” because they did not want to go further on tapering down or stop using medication. This package includes physical activity, recovery workshop, art therapy, network meetings – and being part of a fellowship. The employees are described as optimistic, willing to share personal experiences and to take part in activities. There are expressions of complementarity in how formal expertise is combined with a willingness to facilitate cooperation between the patient, family and services.

It is interesting how they express being satisfied or dissatisfied. Some explicitly express a kind of debt of gratitude, that they feel they have to do what is possible to give other people the same opportunity. There is an awareness of the uniqueness in getting this treatment and somehow being part of an exclusive group; “*an experience of getting a chance to drive a luxury car instead of an ordinary plain car”* as expressed by one participant [13].

However, reciprocity between staff and patients is not uncomplicated. We are relying on patients’ descriptions on the content and degree of sharing, without attempting to validate these accounts against the staff’s reports. While some participants value the reciprocity positively, others express that it leads to uncertainty. Reciprocity among fellow patients, as mentioned by Ahmed [14], is valued in the present study as one of the most important factors and a source of knowledge and personal growth.

### Frames and content

A stable network and a professional engagement stimulate bonding and bridging [19]. The dialogue between community and specialist health care along with in some cases, limited access to care, are vulnerable factors to ensure continued progress. Sufficient time for exploration is described as crucial.

Sharing experiences from treatment reflects on what they received, but just as much on how it was. While this might be self-evident, it touches the core in how we understand the data. The treatment content is of importance, but human encounters are decisive. Zeitgeist, purpose and availability are all experienced as influential on their own definition of outcome, such as quality of life, worries or satisfaction with life. This may indicate that what one wants help for can often be opposed to the treatment offered [20–22]. For example managing symptoms without medication as opposed to reducing symptoms through medication and other interventions.

### What is it all about?

What differentiates and defines the perception of “success” or “failure” is not clear-cut. While external objectives for the establishment of the ward may be met, the question of what “medication free treatment” truly means, remains unanswered. When we listen to the participants’ stories, the experiences are not unanimous. The data describe a focus on individual motivation, mobilization and active adaption. While everyone acknowledges the process, not everyone describes fulfilment or achievement of their own wishes, or of the ward’s goal of tapering off. Many participants reflect on why things did not work out as they hoped; citing factors such as a general ambivalence towards tapering off, a profound fear of doing so, or concerns about having given overly negative feedback to the ward. When the participants do manage to taper off, they strongly validate the ward’s competence, highlighting the positive environment, the hard work involved and their own strong motivation. A belief in change and maintaining hope are active and positive strategies. Building an alliance, through using time and being flexible, makes it possible to gain trust, even in the face of a history of negative experiences and insecurities. In line with the description of Ødegaard et al. on self-motivation [25], participants explicitly describe themselves as active agents in their processes, especially when it comes to their wishes and decisions.

### Strengths and limitations

The qualitative design and the selection of participants who specifically sought this treatment option prevent us from discussing any kind of treatment effect, or aspects connected to medication dosages.

The sampling of participants was based on those who responded to the invitation which may have introduced a selection bias regarding experiences, though the direction of this bias is unclear. Many are aware of the political struggle leading to the establishment of the ward unit. Some might participate because they feel they are in debt to the ward, and that they are obliged to support anything that can give the ward credit. This may have dampened negative statements. Despite this concern, the high number of participants, the presence of diverse experiences and opinions among the participants and the continuous and dynamic evaluation of possible themes from the first interview suggest that this issue was effectively mitigated. In our opinion, the 19 persons hence represent a valid variety of experiences, and the processing and piloting of the questions strengthens our confidence in content validity.

Data reliability was ensured through detailed transcription of all interviews and data organization using NVivo, facilitating accessibility and analytical clarity. The research team had full access to the data, maintaining transparency. We also discussed our interpretations with a diverse reference group, further bolstering the credibility and confirmability of our findings.

Nevertheless, the potential for biases stemming from the researchers’ professional backgrounds and clinical experiences remains. The interviewer is a psychologist employed at the hospital, possibly affecting participant openness and the researchers’ interpretations due to known or unknown biases.

The study’s unique setting limits its transferability. However, it contributes general insights potentially applicable to other areas, particularly other sectors of mental health care dealing with severe mental illness.

## Conclusion

Participation in an ongoing dialogue on medication, personal experiences and recovery are important factors in how the participants describe the medication free ward. Three concepts are identified as crucial: Tapering off, Relations, and Frames and content. A fourth concept describes overarching aspects that connect the first three. With the label «medication free treatment», the significance of gradually discontinuing medication is evident. For many, this determines whether they are eligible for the treatment. Often, the inability to taper down is attributed to intrapersonal factors such as not being ready or needing something different. Those who have successfully tapered down often recognize their own dedication, but their narratives also highlight the influence of the ward’s frames and values. There is a sense of being part of something new and «exclusive». However, the therapeutic approach does not seem to introduce entirely new concepts but rather emphasizes well-known elements such as the importance of being seen, and the value of meaningful experiences. Moreover, «medication free» is more complex than it sounds; many continue to use some kind of medication. The participants’ descriptions perhaps might reveal more about what they felt were lacking in their previous treatment course. This suggests to reemphasize the importance of empowerment and empathetic relationships in treating severe mental illness, to foster a sense of coherence and meaningfulness.

What we observe in this study is not just imagined but real experiences from processes where the importance of medication is downplayed. The results hence contribute to nuancing the debate on medication free treatment and also provide a basis for understanding patients’ need for help and their motivation. Additionally, the results contribute to understanding what “medication free” can represent beyond just the direct act of taking/not taking medications, and what patients find helpful when trying to cope with severe mental illness.

## Supplementary Information

Supplementary Material 1.

Supplementary Material 2.

Supplementary Material 3.

Supplementary Material 4.

## Data Availability

The dataset used and/or analysed during the current study consists of in-depth qualitative patient interviews not publicaly available for confidentiality reasons. Information about the dataset are available from corresponding author on reasonable request. The interview guide is available as an supplementary file to earlier publication [27].

## Abbreviations

CHIME: Model of personal recovery based on connectedness, hope and optimism, identity, meaning and empowerment
DPS: Nidelv District Psychiatric Centre, St Olav Hospital, Trondheim Norway
ICD-10: International Classification of Diseases, 10th Revision
IMR: Illness Management and Recovery
KBT: Competence Centre for Lived Experience and Service Development
NTNU: The Norwegian University of Science and Technology
NVivo: Software supporting qualitative and mixed methods studies
RW: Recovery workshop
STC: Malterud Systematic Text Condensation
UiT: UiT The Arctic University of Norway
UNN: University Hospital of North Norway
