# Supplementary material for: What is it all about? An explorative study of patients’ experiences with medication free treatment
Source: BMC Psychiatry. 2024 Dec 2;24:872. doi: 10.1186/s12888-024-06327-5 (PMC11613473; doi:10.1186/s12888-024-06327-5)
Supplement: Supplementary file 1 — Supplementary Material 1. [file 12888_2024_6327_MOESM1_ESM.pdf]

Medication Free Treatment Programme

Treatment without the use of neuroleptics for people with severe mental disorders at the University Hospital of Northern Norway

Contents

Introduction 2
How the Norwegian health care system is organized 3
Reasons for offering medication free services in Norway 6
Development of Medication Free Treatment at University Hospital of Northern Norway ... 8
Course of treatment, medication free treatment 11
Recovery-promoting measures in the treatment.. 14
Treatment content at the unit 17
Medications – reduction – tapering strips 23
Experiences thus far 26
References.. 28

Introduction

The University Hospital in Northern Norway saw considerable national and international attention regarding the establishment of a medication free treatment programme at the hospital in January 2017. This is probably due to several factors, but not least was that there were no offers within the framework of traditional public psychiatric hospitals or clinics anywhere else around the world.

The unit experienced a number of enquiries from the start, from users of mental health services and professionals who wanted to visit the unit. Some just came to see, while others came to consider the possibility of cooperating with the unit in various ways.

In December 2019, the head of the psychiatric association in Lithuania, Ramune Mazaliauskiene, contacted the unit asking if two of his colleagues could visit to explore the medication free treatment programme. In planning this visit, it quickly became clear that there was a desire among colleagues from Lithuania to bring about a concrete collaboration on a project that could provide the basis for applying for funding from the Active Citizens Fund (https://apf.lt/en/dvisalis-bendradarbiavimas/. Funding from this fund presupposes a bilateral cooperation between an "implementing partner" in Lithuania and a "partner" in one of the "donor countries" (Iceland, Liechtenstein and Norway). The project they wanted to collaborate on was called "Wings of Change", promoting human rights and social inclusion of people with intellectual and/or functional disabilities in 10 regions of Lithuania.

As a "partner" in this project, the medication free treatment unit mainly committed to participating via collaboration meetings/consultations with the "implementing partners" in Lithuania, in addition to drawing up a written description of the practice developed in the medication free treatment programme.

Tromsø, June 2021

How the Norwegian health care system is organized

In Norway, public social security and social insurance are called the National Insurance Scheme. You must be a member of the National Insurance Scheme to be entitled to benefits under the National Insurance Act. As a member of the Scheme, you get access to all public health and care services. As a general rule, everyone who resides or works legally in Norway is a member of the National Insurance Scheme. This means the vast majority of people living in Norway have access to free and equal health services, regardless of place of residence and income.

The Norwegian health system is partially decentralised. The responsibility for specialist treatment lies with the state. There are four regional health trusts around the country, one for each major geographical region, that are responsible for providing specialist health services for the population of their health region. They also own the public hospitals in the region. The public hospitals are organized as *health trusts*, which are managed by the regional health trusts.

The municipalities are responsible for the primary health and care services. In primary health care, the general practitioner is the patient's most important and first contact with the health services. Everyone has the right to be assigned or choose a family doctor. The general practitioner offers daytime health services for people on his patient list. This also includes emergency help. The general practitioner also cooperates with other services within primary health and care services and the social services when there is a need for such cooperation with regard to the residents on their lists. The collaboration can include home-based services, nursing homes for admission and discharge, child health clinics and health stations, municipal drug addiction care services etc. The general

practitioner plays an important role in coordinating the patient's need for medical services.

Depending on the patient's need for treatment or help, a general pratitioner can refer a patient for necessary health care. A patient who needs to be examined and/or treated by a *specialist health service* for an illness can be referred to a specialist. These can be private contract specialists, specialists with hospice expertise at a district medical center, at a decentralised outpatient clinic or specialists at outpatient clinics at hospitals. A person in need of treatment within mental health care may be referred to a district psychiatric center. These offer specialised services in mental health care and can also receive patients for daytime treatment or hospitalisation. People with serious and acute psychiatric conditions are admitted to emergency departments at hospitals. Patients with substance abuse problems may be referred for outpatient treatment or hospitalisation at a substance abuse institution.

For conditions (both mental and somatic) that require hospitalisation, the patient is referred to a hospital. The patient can choose which hospital he/she prefers through a scheme called *free hospital choice*, and they can also choose to be treated at a private hospital that has an agreement with the regional health authorities. Due to their medical specialisations, the hospitals often become more specialised, and the tasks are often shared between the hospitals.

Treatment of mental disorders is voluntary, as a general rule. Patients who are admitted to an institution have the right to leave the institution whenever they wish. As a general rule, patients who are admitted voluntarily cannot be medicated without their consent. The Mental Health Care Act also allows for patients to be subject to compulsory mental health care when certain conditions are met. This can be carried out in three ways:

inpatient, or outpatient with compulsory treatment, and in addition compulsory observation which means the patient can be detained in the hospital for up to 10 days so the hospital can assess conditions for compulsory mental health care. Pursuant to the law, decisions can also be made on more intrusive measures such as protection, coercive measures and coercive medication.

Medication Free Treatment is part of the specialist health services, i.e. the state's responsibility. Patients who want treatment at the unit therefore must be referred by a therapist in the outpatient department of a specialist health service, most often from a district psychiatric center. This presupposes a referral from a general practitioner. This means that in order to receive treatment at our unit, you must live in Norway. Our Medication Free Treatment Programme is voluntary. This means the patient him/herself must want treatment, and the patient has the right to end treatment whenever he wants.

Reasons for offering medication free services in Norway

In 2015, the Ministry of Health and Care Services (HoD) sent out a document in which they instructed all regional health trusts in Norway to establish their own medication free units (HoD, 2015). The demand came, among other things, after several user organizations had joined forces in a collaboration they called the Joint Action for Medication Free Treatment (Fellesaksjonen, 2013). The goal for this collaboration was for real medication free treatment alternatives to be available from public specialist health services, especially for people with severe mental illness. The user organizations explained their experiences and suggested that people with psychosis and bipolar disorder in particular experienced pressure to take neuroleptics, and that there were few real opportunities for other treatment and help to reduce it. The basic document issued by the Joint Action stated that people who want medication free treatment often

had no inpatient services available in the mental hospitals, and that this should be available on the basis of their freedom to choose treatment they themselves that they believe in and want.
The joint action called for setting up services where people were not subjected to compulsory medication or pressure to take medication, where basic treatment was a safe place to be, and to gather experience to develop good treatment cultures for nondrug treatment that could strengthen the field's need for knowledge development.

Medication Free Treatment offered by the University Hospital of Northern Norway opened in January 2017. The debate that followed around medication free treatment of psychosis and bipolar disorder has been extensive and interesting to follow. It has created commitment in many areas. Key topics have revolved around issues related to the knowledge base for efficacy and risk related especially to long-term treatment with neuroleptics and the professional soundness of medication free treatment for psychosis and bipolar disorder. Regarding professional soundness, the Norwegian Directorate of Health (2017) has specified that if the patient is competent to give consent, treatment should be voluntary and the patient thus has the right to participate in choosing between sound treatment options. What professionally sound means, is an assessment that includes factors about the patient, the treatment offered and the circumstances of the situation. While medication free treatment was getting started in Norway, the debate initially appeared to be polarized on different opinions about what is the best treatment for serious mental illness. Some thought that medication free treatment was unjustifiable, experimental and thoughtless. Gradually, there was a growing acceptance that there is evidence for different treatment approaches, and that the introduction of

medication free treatment options primarily concerned a patient's right to autonomy, participation and choosing over their own lives.

Medication free treatment is thus more about human rights, user participation and the right to make informed choices about one's own treatment, than about the effect of different treatment approaches.

When the Minister of Health Bent Høie cut the cord at the official opening of MFBT on 31 January 2017, he said that the introduction of medication free services in the public health service in Norway would help to make patient health services even better by meeting the needs of the patients themselves. Now they could be considered experts on their own health and decide which treatment they wanted, even if this does not involve medication with neuroleptics.

Development of Medication Free Treatment (MFBT) at University Hospital of Northern Norway (UNN)

Following the request from HoD, a protocol group was established at the northernmost health trust, consisting of representatives from various user organizations and professionals from UNN. The protocol established a framework for medication-free options, which was described in the following main features:

- The medication free option was to be established as a inpatient unit with 4-6 patient beds. The offer was to be a regional offer for the Northern Norway Health Trust (Helse Nord), and the target group was to be people with severe mental illness, primarily psychosis or bipolar disorder, who wanted medication free treatment.

- The medication free unit was to be based on Norwegian health legislation and national professional guidelines – in line with other treatment options in the specialist health services.
- The offer should be based on the Joint Action's basic ideas
- The offer was to increase the freedom of choice for these patients and be part of a larger investment in medication free treatment and reduction of unnecessary use of drugs in mental health care, and be based on voluntariness.
- Run as a recovery-based option

After the framework was approved, it was up to the unit itself to develop the programme further. The assignment from HoD stated that the offer should be designed in close collaboration with user organizations; MFBT solved this by establishing a collaboration group that consisted of employees from MFBT and representatives from various user organizations. The collaboration group met regularly the first year, and had an advisory function in the development of the treatment offer. For MFBT, it was important to strive for the integration of the basic ideas from the Joint Action (Fellesaksjonen 2013) with Norwegian health legislation and national professional guidelines as the legal basis (Norwegian Directorate of Health, 2013) in line with other treatment options within the specialist health services. It was decided that the Inpatient Department would be located in Tromsø and accommodate 6 inpatients.

The units's personnel organization is similar to most other inpatient units in Norway, with a staffing factor similar to an emergency unit. There are 24 employees at the unit who are trained nurses, occupational therapists, social workers, occupational therapists physiotherapists, milieu therapists, experience consultants, psychologists, doctors and chief physicians. Most people who work at the unit have different higher educations or

are still studying higher education in different fields. There is therefore also expertise in various therapeutic directions: art and expression therapy, individual Placement and Support (IPS) and psychomotor/physical understanding. An experience consultant is employed in the unit; this position is also part of the unit's management team. This has been useful for a basic development of an understanding of experiential competence at the unit, which has contributed to a practice that transcends "user participation". From the user organizations, experience competence among employees was desirable. In the recruitment process when the programme was being set up, personal experience of employees was considered a valued competence.

To work at the unit, there was a prerequisite that the employees believe medication free treatment has a value in itself, and that they want to work with just that. The prerequisite for this was to establish a completely new department where everyone who wanted to work there had to apply. This contributed to establishing a completely new professional environment within medication free treatment, and also an important *spearhead* in the work of session experience and increasing the knowledge base. Elsewhere in Norway, this was not possible, as other hospitals chose to solve the assignment from HoD differently; e.g., by dedicating one bed in an already existing inpatient unit to drug-free treatment.

Course of treatment, medication free treatment

Course of treatment. This is a *course of treatment*, which consists of collaboration between several agencies and often several admissions at MBFT over time. The district psychiatric center and municipal services run the primary care side of the programme. A private network and team at MFBT will support the programme, in the form of admissions and collaboration via network meetings, guidance, etc.

This means medication free treatment is not only something that happens with a limited admission to the hospital unit, but simultaneously in several parts of the patient treatment network.

This enables long-term processes, preferably over several years, without the patient having to be hospitalised during the period. Through network meetings in the period

between referral and first admission to MFBT, an overall comprehensive treatment plan is offered in collaboration with the network, where admissions to MFBT are part of the process and what treatment is offered. Frequency, length and treatment focus at admission vary based on the patient's wishes and needs, and what one arrives at in dialogue within the network. An average course lasts about 2-3 years and consists of outpatient treatment at the district psychiatric center and 4-6 planned admissions per year at the unit for post drug-free treatment. A flexible approach is sought so that, as far as possible, it is not the patients who have to adapt to MFBT, but MFBT try to adapt to each individual patient.

Patients who apply for MFBT want and believe that recovery is possible without the use of neuroleptics. Many patients need assistance in tapering medications after long-term use of neuroleptics. The treatment goal for patients is often the opportunity to explore other ways of working with and dealing with their mental and social challenges. This often involves raising awareness about processes that lead to challenges, as well as exploring and testing alternative ways of dealing with this. As life must be lived at home and in one's own immediate environment, this is also a key factor in the programme being organized as a process. It is at home that life is lived, where you experience challenges when tapering or stopping medications. New ways of behaving must therefore also be anchored in the process and tested out in this environment. Recovery is also a social process, even though it feels personal and individual. That is why it is important to have good support from your local network when embarking on a longterm project such as managing to live without medication when struggling with psychosis or bipolar disorders.

The protocol group described how the treatment should be based on values related specifically to recovery and be based on relationships and networks.

Recovery anchoring. Our understanding of the concept of recovery is that – in a recoverybased health service – the focus shifts from illness to resources and opportunities. This means more than emphasising the latter.

MFBT's understanding of the concept of recovery within a relational and network perspective means that various mental conditions are understood more as reactions to lived life than as medical conditions, and that the treatment is about learning to deal with various challenges as opposed to reducing, alleviating and curbing symptoms.

Changing one's relationship to what is happening, as opposed to controlling what is happening, is a key change mechanism in line with most recent psychotherapeutic treatment methods such as Acceptance and Commitment Therapy (Hayes, Strosahl and Wilson, 2011) and metacognitive therapy (Wells, 2007). This is anchored in the patient's everyday life and network, by involving important relationships in the process of the individual patient.

From a relationship and network perspective. Getting by without medication when you have a serious mental illness is often a long and demanding process that requires individual adaptation. It is important that the treatment approach reflects this. The starting point for MFBT has therefore been that when a person is referred to the unit, the treatment offer is created in dialogue between the person concerned and important people in his or her life. From a relational and networking perspective, mental disorders are not something that occur inside a person, but between people. That is, mental challenges are often experienced in meaningful relationships. This is the reason why,

within this perspective, emphasis is placed on seeking understanding and solutions to the challenges of relationships. The approach involves collaborating with each patient's network. Networking approaches help create belonging and community around something that is close to the patient's life. Belonging and a sense of community arise when people who are important to each other come together if something has a huge driving force in recovery processes. These factors contribute to the reduction of difference, exclusion, stigma, reduced autonomy, existential anxiety and shame, which in many cases prove to be key challenges for patients in the course of MFBT.

Recovery-promoting measures in the treatment

The unit has placed emphasis on facilitating measures and actions that are in line with recovery values, and that can promote sustainable change processes. Very many of the patients who are on a medication free course of treatment have previously experienced compulsory treatment in mental health care, both in the form of hospitalisations and as compulsory medication. Experience from the unit shows that measures where the patient's autonomy is promoted in a fundamental way are of great importance in improvement processes. The rest of this document describes our key practices in medicine free treatment.

*Self-referral***.** Our patients are asked to write a self-referral, where the purpose from the start is to bring out the patient's thoughts and opinions. This emphasises that MFBT is a voluntary treatment programme that requires motivation and commitment from the patient. Furthermore, the self-referral contributes to the patient starting the treatment process at home with their family, therapist and network to anchor the process in their lives, where they live, on a daily basis. In the self-referral, the patient describes the reasons for the desire for medication free treatment, previous experiences with treatment, recovery goals and what interests he or she has.

Network meetings. An initial network meeting is planned after the referral has been received and it has been assessed that the patient falls within the target group for medication free treatment. The patient is referred to the meeting and asked to join, along with others whom the patient wants to include. This can be family members, follow-up services, the family doctor or others who have an important place in the patient's network. Throughout the process, regular network meetings are held to ensure good collaboration and follow-up around the treatment of each patient. A network meeting is more than a traditional collaboration meeting where different tasks are distributed and information is shared. Network meetings are, among other things, a central and important arena in the treatment processes where a common understanding of the recovery process is established by the group, and important decisions are made, often with the use of reflective conversations or elements known from open dialogue practices.

Team meetings. Each patient gets his or her own team at MFBT. This team consists of 3 employees at MFBT in addition to the patient himself. The doctor/chief physician

participates in the team meetings if there is a need for it when they themselves are not a therapist in the relevant team. During the periods when the patient is hospitalised, a team meeting is held approximately once a week. The patient always participates in a team meeting. The purpose of the meeting is to plan, evaluate and make adjustments to the admission, as well as have a dialogue about the team that the patient or other team member thinks is important.

Nothing about me without me. The unit strives to not have conversations or meetings about the patient that involve making decisions, or understanding/interpreting the patient's behaviour/condition, without the patient being present. This means that the patient participates in all of the treatment meetings etc. that deal with the patient himself.

Self-documentation. Norwegian health personnel follow strict requirements for documentation and record keeping of treatments. Both a continuous environmental journal and a case summary are written for treatment. Self-documentation is an offer the patient receives to write their own medical notes in the same way as health personnel. The note that the patient writes himself becomes part of the patient's records, together with other notes. Patients at the hospital have full access to their own medical records and can log in via an electronic solution and continuously read the medical records that are written about them by healthcare professionals.

Reflective conversations.

In some processes, reflective conversations are used, a method developed by psychiatrist Tom Andersen in the 1980s. The reason for developing this method was a

comprehensive focus on the pathological, and a desire to develop an approach that emphasised interpersonal recognition. The method means that the therapists do not take on an expert role with a final answer, but rather to facilitate for the patients themselves to find solutions by participating in open dialogue, reflection and being in a listening position. This is consistent with our treatment, and means that the patient always has the role of the expert in his or her own life, while the therapist's role is as much as possible about supporting processes where the patient can explore, experience, make choices and take steps in line with their values.

Treatment content at the unit

Medication free services shall be based on national guidelines (Norwegian Directorate of Health, 2013), which recommend measures such as conversation therapy, family collaboration, music therapy, physical activity, social skills training and measures aimed at helping the patient in work or education. The treatment at the MFBT inpatient unit has been developed in line with these guidelines, based on daily group activities consisting of physical activity, art therapy and Recovery Workshops. The patient can also, if necessary, receive individual treatment measures from an experienced consultant, psychologist, physiotherapist, etc. The unit is structured and set up as a working day, with treatment from morning until dinner. The planned admissions to the unit provide an opportunity to work therapeutically with specific challenges. The

environment at the inpatient unit is considered an arena for receiving support/guidance to work with individual recovery goals.

Weekly Programme. The treatment programme at MFBT is based on planned group activities, groups and meals happen together, at the same time as treatment is individually adapted to the patient and there are opportunities to do something else if a group or activity seems too demanding. The unit is structured around a culture and an environment where everyone is together, both patients and staff. This is done to facilitate the creation of good and equal experiences, conversations and experiences. There is also room to be alone with oneself and choose to be social according to what one feels one can master. The treatment programme contributes to the structure of everyday life at the unit and can be a starting point for exploring what the patient can acquire and use in everyday life at home. All the activities on the schedule can function as an arena where the patient can explore and practice individual recovery goals, and be set up within networks or teams. For example, a patient may desire to speak in a group, practice concentration, have something to stand up for, improve physical health or practice being in a conversation with others while experiencing auditory hallucinations.

The weekly programme contains 5 different groups/activities: Recovery Workshop, mindfulness, creative workshop, recovery through music and physical activity.

	Monday	Tuesday	Wednesday	Thursday	Friday	Saturday	Sunday
08.00	Breakfast	Breakfast	Breakfast	Breakfast	Breakfast		
08.15	Employees meeting	Employees meeting	Employees meeting	Employees meeting	Employees meeting		
08.45	Daily meeting	Daily meeting	Daily meeting	Daily meeting	Daily meeting		
08.55			Whiteboard meeting				
09.05	Mindfullnes exs.	Mindfullnes exs.	Mindfullnes exs.	Mindfullnes exs.	Mindfullnes exs.		
09.30	Physical exersice group	Recovery workshop group	Physical exersice group		Physical exersice group		
10.00							
10.30							
11.00	Lunch	Lunch	Lunch	Lunch	Lunch		
11.30							
12.00		Art therapy group	Recovery workshop group	Recovery workshop group	Recovery workshop group		
12.30							
13.00							
13.30							
14.00							
14.30		Intern teaching	Guidance		Sweet end of the week		
15.00							
15.30							
16.00	Dinner	Dinner	Dinner	Dinner	Dinner	Dinner	Dinner
16.30							
17.00							
17.30							
18.00							
18.30							
19.30	Supper	Supper	Supper	Supper	Supper	Supper	Supper
20.00							
20.30							
21.00							

Recovery workshop. The Recovery Workshop is a group-based treatment one hour four days per week. It is a group in which both patients and staff participate. The purpose of the Recovery Workshop is to increase awareness of one's own health and life situation, which increases the probability of perceived mastery and self-care. Self-management and self-care are key to the recovery perspective, where the goal is to give people the opportunity to seize their own process or regain control of their own lives.

For each week, there is one recurring theme for the four sessions. In the first session, this theme is linked to the concept of recovery and recovery processes. Recovery is both a personal and a social process and involves working with hopes and dreams to live a meaningful life. Here the focus is on coping with mental disorders and what one can do to be better, with oneself, and in the context in which individual lives are lived. In the Recovery Workshop, this is done through a combination of teaching, group reflection, dialogue and sharing different forms of knowledge.

The Recovery Workshop takes its inspiration from the IMR programme (Mueser, 2006) and many of the themes in the Recovery Workshop coincide with those found in the IMR programme. However, dialogue and sharing of experience are largely facilitated in this offer, more than the dissemination of knowledge through traditional teaching and lecturing. The whiteboards are used extensively to keep focus and reflect together on the week's theme. Compared to the IMR programme, our Recovery Workshop is not manual-based and structured, and it places greater emphasis on dialogue and group reflection in the here-and-now than on individual tasks during the sessions and between the sessions. The experiential knowledge that both patients and employees bring to the workshop is emphasised, valued and given a lot of space. In this way, the Recovery Workshop is probably more akin to Recovery College as it was developed with origins in England (Perkins, 2018). At Recovery College, emphasis is placed on experiential knowledge on an equal footing with theoretical knowledge. Those who participate in the courses are participants and not patients, they are not referred, but register themselves for the courses. The courses offered at Recovery College are relevant to people in general and focus on how everyday life can be lived with the challenges the individual has. In the IMR programme, a traditional understanding of illness is to a greater extent the basis for approach and focus, with a stronger emphasis on understanding illness and symptom management.

Experiential knowledge has a large place in our Recovery Workshop and it is encouraged that those present, both patients and employees, share their thoughts and experiences related to the relevant topics. The themes are, with some exceptions, largely universal and commonplace. Examples of such topics are: Sleep, Diet, Physical Activity, Everyday Life and Coping, Dreams and Responsibilities, What is Normality? Coping and meaning, Anxiety, freedom and life, How to take care of oneself, My flock, Getting to know oneself,

Emotions and emotion regulation, Spirituality, faith and existence. Examples of more specific topics are: Hearing voices, Medications and Mental disorders, Crises and coping plans, Shared reading.

Mindfulness. Mindfulness is on the programme every day from 09:05-09:20. The group is led by an employee. The purpose of the group is to get to know one's own breathing, become confident in sitting in a group with closed eyes, get to know one's feelings and thoughts in an accepting/mindful way, and to experience one's own body and the connection it has with both breath, emotions, thoughts and surroundings. After each session, everyone says a little about how it felt to be in the group – if they want to – and we talk a little about what will happen that day.

Creative group. The unit has a creative group once a week, and the group is led by an employee with an education in art and expression therapy.
Art therapy is to express oneself through creativity. Creating an expression, such as an image, can be a way to put "words" into thoughts and feelings. When you create something with your hands, words are not needed, the words come afterwards. Pictures, colours and symbols can tell a story. Creating can help you discover resources you did not know existed. The images can also be relieving by expressing something you did not know you had inside you. It does not matter what you create or whether you are good at drawing.

Recovery through music. This group is held once a week and is led by a music therapist. Music is the starting point for this activity session. You listen to music suggested by the

leader of the group or at the request of participants in the group, and you share your thoughts and feelings about what the music means or does with an individual.

Physical activity. The programme includes physical exercise, in groups, planned 3 days a week. The exercise is arranged so that everyone can participate. 2 of the days the training takes place in the gymnasium as strength training. 1 of the days is set up as outdoor cardio training.

After dinner there is free time in the unit. The employees who are at work are available for the patients who are admitted and offer activities, conversations and follow-up based on needs and treatment plan. The unit wants to be a friendly place and always has different projects that take place jointly between patients and staff. These can be small everyday activities such as walks in the mountains and fields, ice swimming, concerts and cinema, but also larger projects such as arranging a music festival for the entire hospital and building a Sami lean-to. The purpose is to create arenas for toghetherness and mastery by testing out new things and experiencing new things. Such activities are relationship-building, both between patients and staff, but also between patients.

Medications – reduction – tapering strips

Medication free treatment is offered to people with severe mental disorders who want a medication free treatment. About half of the patients use medication during hospitalisation. These patients want help to reduce their neuroleptics (or so-called mood-stabilizing drugs). There is a lack of knowledge on maintenance treatment with neuroletics beyond 2 years (Harrow et al., 2013, 2021). Despite this, many patients remain on these drugs for many years. Similarly, knowledge about the reduction/tapering of medications used for psychosis and bipolar disorder is also very deficient. In the training of psychiatrists and health professionals, training in the reduction of these medicines is completely absent. Gradually reducing these drugs, especially after long-term use, is far more problematic than what prescription

instructions, professional guidelines or professional literature describes. Among other things, completely new ailments can appear during attempts at reducing such medicines.

The user organizations and interest groups for people with mental illness, however, have known this for a long time. This has meant that the users of these medicines have taken various initiatives to gather and disseminate experiential knowledge on how to gradually reduce their medication. One example of this is the "Harm reduction guidelines to quit or use less psychotropic drugs" which was first published in 2007 in English by Will Hall and The Icarus Project. These guidelines were translated into a number of languages as a collection of people's experiences and practical advice for reducing or tapering psychotropic drugs. "The Inner Compass Initiative" is an example of a website run by people with their own experiences from reducing their use of psychotropic drugs. Here you will find advice on how to proceed when phasing out medicines, including concrete and detailed descriptions of how you can modify medication doses yourself. Both of these initiatives are examples of how users themselves have gathered and made knowledge available when this has been manifold in the field in general.

The Norwegian Psychiatric Association published Clinical advice for phasing out and discontinuation of antipsychotic drugs in 2020. Here they acknowledged that tapering and discontinuation of so-called antipsychotic drugs can be very demanding and that there is limited knowledge about this process. Here is also a list of tapering symptoms and recommendations to map these during tapering. A tapering step of 10-25% of the initial dose every 2-3 months is recommended.

An article published in 2021 by Horowitz et al. describes a method for reducing neuroleptics. Here it is suggested that antipsychotics should be reduced with a smaller and smaller dose reduction the closer one gets to complete discontinuation. It proposes a 5-10% reduction of the last dose of antipsychotics every 3-6 months. The chances of withdrawal symptoms are reduced using such a strategy. The last dose before discontinuation may then be as small as 1/40 of the original dose. In order to be able to implement such a reduction regimen, there will be a need for medication in far more and smaller doses than what is currently available through ordinary doses in drugstores.

In the Netherlands, in a collaboration between patients, researchers and a pharmacist, *tapering strips* have been developed that enable a far more gradual reduction of psychoactive medicines than ordinary medicine doses allow. Since the first tapering strip was available in 2013, this solution is currently available for 49 different psychoactive drugs. For a short period in 2019-20, it was possible to import tapering strips to Norway. However, this possibility of import was stopped by the Norwegian Medicines Agency in 2020, allegedly due to an export ban in the Netherlands. Access to this solution, or strips similarly produced in Norway, is absolutely necessary for more people to succeed in phasing out psychotropic drugs.

Elements that the unit considers for tapering:

- What previous experiences does the patient have with tapering and discontinuation?
- Timing. Is this the right time to start a reduction? Is life otherwise stable in terms of housing, finances, networks and relationships, activity, education or work?
- Is there support in your own network, both private and public, for tapering?

- Has an emergency plan been prepared that describes possible deterioration and what can be done to prevent and deal with this? What ailments can be expected to appear? Will the ailments that initially led to the start of the medication reappear? In this case, how should this be handled without medication?
- At what speed should tapering occur? Prepare for possible challenges and symptoms/new ailments and the need for breaks in tapering and adjustment to the reduction plan.
- How should one's basic needs for nutrition, sleep, rest, exercise be attended to?
- Considering the needs of a place to live, work or money to live on, something to do, someone to do it with.

Experiences thus far

The medication free treatment programme has now been in operation for almost 5 years. At the time of writing, approximately 80 people have received treatment courses at the unit. The organization has the possibility of running around 30 different active patient courses, where people in such treatment courses also receive treatment from services at their local district psychiatric center and at planned admissions to the unit.

2/3 of those who come to the unit are women, 1/3 are men. The age range has varied between 19 and 63 years. The number of referrals has been fairly evenly distributed over the years in which the programme has existed. Over half of those who are referred have a persistent psychosis problem as a reason for referral, the rest have a diagnosis within the bipolar spectrum. The vast majority have other additional diagnoses. People

with ongoing drug addiction are not offered treatment. Virtually everyone who has been in the process has had previous admissions to mental health care in Norway, a very high proportion have compulsory experiences, both in the form of involuntary admissions and compulsory medication with neuroleptics.

As the course is created and established on the basis of what is best for the individual patient, there is great variation in the number of admissions and the length and content of the admission. The number of admissions has thus varied between 1 and 26, and the length of the admission has varied between 1 day and 1 year. The most common, however, are planned admissions of 2 weeks, 1-3 times in six months during the period you are in an active course of treatment.

In relation to the use and tapering of neuropleptics, almost all patients who have come to the unit have previous experience with the use of neuroleptics. At the time of referral, approximately half have used neuroleptics regularly, while the remaining have not used neuroleptics regularly. Descriptive for the latter group is that the vast majority had previous experience with neuroleptic use, but had discontinued on their own before admission. The remaining group wanted assistance from the unit to reduce their medications. Our experience shows that tapering and being able to live without medication, especially when you have used medication for a long time, can be a very demanding and lengthy process. Especially in situations where a person uses several different types of neuroleptics, the tapering process can extend over several years. Slow tapering can reduce the risk of tapering symptoms. But perhaps most important are the psychological processes that take place in connection with painful and demanding inner experiences perhaps becoming more accessible, and sought to be handled in other ways without the use of medication.

Medication free treatment is available, but it is still in a developmental phase. Collaboration with the patients and their networks that have taken place at the unit has contributed to an increase in professional experience and competence. Experiences are gathered along the way, mainly via dialogue, and are used for continuous development with the patient in focus. We hope that the treatment programme can meet the expectations for the unit contributing to better knowledge and further disseminating such experiences. The unit prioritises participating in dissemination of the treatment and experiences in various professional forums, in addition to various dialogues with interest organizations and international organizations. There is great international interest in medication free treatment, also in the general media.

References

Fellesaksjonen (2013, 11. februar). *Grunndokument for Fellesaksjonen for medisinfrie behandlingsforløp i psykiske helsetjenester*. https://medisinfrietilbud.no/grunndokument/

Gøtzshe, P.C. (2015). *Dødelig psykiatri og organisert fornektelse.* Oslo: Abstrakt forlag.

Hall, W. (2014). *Skadereduserende veileder for å slutte med eller bruke mindre psykofarmaka.* Nasjonalt senter for erfaringskompetanse innen psykisk helse. *Harm reduction guide to coming off psychiatric drugs* (2nd ed., p. 51). (2012). https://willhall.net/comingoffmeds/ Creative commons.

Harrow, M. and T. H. Jobe (2013). "Does Long-Term Treatment of Schizophrenia With Antipsychotic Medications Facilitate Recovery?" Schizophr Bull **39**(5): 962-965.

Harrow, M., et al. (2021). "Twenty-year effects of antipsychotics in schizophrenia and affective psychotic disorders." Psychol Med: 1-11.

Helsedirektoratet. (2017). *Svar på henvendelse – Faglig forsvarlighet ved legemiddelfrie behandlingstilbud.* https://www.helsedirektoratet.no/tema/psykiskhelsevernloven/Faglig%20forsv

arlighet%20ved%20legemiddelfrie%20behandlingstilbud%202017.pdf/_/attachment/inline/015a3af7-8784-4072-9784b140d50c255c:c2a49e99f31c5397c7dd7b42a0ab0014080980a5/Faglig%20fors varlighet%20ved%20legemiddelfrie%20behandlingstilbud%202017.pdf

Helsedirektoratet. (2013). *Nasjonal faglig retningslinje for utredning, behandling og oppfølging av personer med psykoselidelser (veileder IS-1957).* https://www.helsedirektoratet.no/retningslinjer/psykoselidelser/Utredning,%20behandling%20og%20oppf%C3%B8lging%20av%20personer%20med%20psykoselidelser%20%E2%80%93%20Nasjonal%20faglig%20retningslinje%20(fullversjon).pdf/_/attachment/inline/a2c5a070-19d8-47df-b86c-9e9e6002c514:643b749f68005e7572f8e70b242c0f0af6f17910/Utredning,%20behandling%20og%20oppf%C3%B8lging%20av%20personer%20med%20psykoselidelser%20%E2%80%93%20Nasjonal%20faglig%20retningslinje%20(fullversjon).pdf

Helse Nord. (2016). *Medikamentfritt behandlingstilbud i psykisk helsevern – innhold og dimensjoner for tilbudet, oppfølging av styresak 143-2015.* https://helse-nord.no/Documents/Styret/Styrem%C3%B8ter/Styrem%C3%B8ter%202016/20160406/Styresak%2042-2016%20Medikamentfritt%20behandlingstilbud%20i%20psykisk%20helsevern.pdf

Helse- og omsorgsdepartementet (2015, 26. november). *Medikamentfrie tilbud i psykisk helsevern - oppfølging av oppdrag 2015.* https://www.regjeringen.no/no/dokumenter/medikamentfrie-tilbud-i-psykiskhelsevern---oppfolging-av-oppdrag-2015/id2464239/

Heyes, S.C., Strosahl, K.D., Wilson, K.G. (2011). *Acceptance and commitment therapy: The process and practice of mindful change.* New York: Guilford Press.

Horowitz, M. A., et al. (2021). "A Method for Tapering Antipsychotic Treatment That May Minimize the Risk of Relapse." Schizophrenia Bulletin.

Moncrieff, J., Cohen, D., Mason, J.P. (2009). The subjective experience of taking antipsychotic medication: a content analysis of internet data. *Acta Psychiatrica Scandinavica.* 120(2)102-111.

Moncrieff, Joanna, Gupta, Swapnil, & Horowitz, Mark Abie. (2020). Barriers to stopping neuroleptic (antipsychotic) treatment in people with schizophrenia, psychosis or bipolar disorder. *Therapeutic Advances in Psychopharmacology*, *10*, 204512532093791–2045125320937910. https://doi.org/10.1177/2045125320937910

Mueser, Kim T, Meyer, Piper S, Penn, David L, Clancy, Richard, Clancy, Donna M, & Salyers, Michelle P. (2006). The illness management and recovery program :

Rationale, development, and preliminary findings. Schizophrenia Bulletin, 32(Supplement 1), S32–S43. https://doi.org/10.1093/schbul/sbl022

Perkins R, Meddings S, Williams S, Repper J (2018) Recovery Colleges 10 Years On, Nottingham, ImROC.

Stendal, Dora Schmidt. (2016). Kan pasientenes egne notater i journalen bidra til å fremme pasientperspektivet i behandlingen? *Tidsskrift for Psykisk Helsearbeid*, *12*(3), 252–262. https://doi.org/10.18261/issn.1504-3010-201603-08)

Taylor, M. J. and S. Yim (2018). "Is there rebound psychosis on withdrawal of antipsychotic medication in schizophrenia?" *Schizophrenia Research, 201*, 430431.

Wells, A. (2007). Cognition about cognition: Metacognitive therapy and change in generalized anxiety disorder and social phobia. *Cognitive and Behavioral Practice, 14*(1), 18-25.

Whitaker, R. (2014). *En psykiatrisk epidemi. Illusjoner om psykiatriske legemidler.* Oslo: Abstrakt forlag.
