# Supplementary material for: What is it all about? An explorative study of patients’ experiences with medication free treatment
Source: BMC Psychiatry. 2024 Dec 2;24:872. doi: 10.1186/s12888-024-06327-5 (PMC11613473; doi:10.1186/s12888-024-06327-5)
Supplement: Supplementary file 2 — Supplementary Material 2. [file 12888_2024_6327_MOESM2_ESM.pdf]

Development of interview guide

In dialogue with Competence Centre for Lived Experience and Service Development (KBT), an interview-guide was developed (appendix xx). After piloting the study in 2017, adjustments were made.
