# Supplementary material for: What is it all about? An explorative study of patients’ experiences with medication free treatment
Source: BMC Psychiatry. 2024 Dec 2;24:872. doi: 10.1186/s12888-024-06327-5 (PMC11613473; doi:10.1186/s12888-024-06327-5)
Supplement: Supplementary file 3 — Supplementary Material 3. [file 12888_2024_6327_MOESM3_ESM.pdf]

Use of medication at the start of treatment and at time of data collection (N=19)

Type of medication	At the start of treatment (N=19)	At the time of interviews* (N= 19)
Antipsychotics	16	14 were still using<br>Registered during treatment course:<br>Of 7 who had tapered down, 3 had to restart<br>Of 2-3 who had reduced, one had to restart<br>4 had made adjustments on type of antipsychotic medication
Mood stabilizers	3	3 (the same persons)<br>1 didn't use during treatment, but was prescribed when acutely admitted later
Antidepressants	4	4 were still using<br>(Not the same persons; two stopped and two others started during the treatment course)
Benzodiazepines (diazepam or oxazepam) or non-benzodiazepine hypnotics (zolpidem or zopiclone)	5-6	10 were still using
Other sleep-related medication (mostly melatonine)	3	3 were still using<br>During treatment course: 1 tried melatonin but returned to zopliclone
Medication for physical conditions**	9	9 were still using<br>2 started during the treatment course
Pain medication	2	3<br>3 started during the treatment course
Vitamines	6	4
No medication	1	2

*14 had finished treatment from the ward and five were still in a treatment course including the ward.
** E.g antihypertensives, hormones, statins, contraceptives.
