# Supplementary material for: What is it all about? An explorative study of patients’ experiences with medication free treatment
Source: BMC Psychiatry. 2024 Dec 2;24:872. doi: 10.1186/s12888-024-06327-5 (PMC11613473; doi:10.1186/s12888-024-06327-5)
Supplement: Supplementary file 4 — Supplementary Material 4. [file 12888_2024_6327_MOESM4_ESM.pdf]

Where the participants heard about the treatment offer

Reception of information	Number of participants
Media	Four said that media was the main source and one expressed ‘having searched for it’ by oneself
Knowing people	Three had heard about the process of establishing the ward through knowing people involved or being politically engaged
Health services	Five had heard about it from a psychologist or psychiatrist. One had information from a relative that had seen an information sheet in a hospital waiting room. One got information from another treatment institution.
Randomly	Three express that they had heard about it randomly, e.g. from a fellow patient or health care worker.
